# Following-Up Micro-Rheological and Microcirculatory Alterations During the Early Wound Healing Phase of Local and Rotated Musculocutaneous Flaps in Rats

**DOI:** 10.3390/life15091424

**Published:** 2025-09-11

**Authors:** Gergo Kincses, Laszlo Adam Fazekas, Adam Varga, Adam Attila Matrai, Nguyen Xuan Loc, Kincso Barabasi, Anna Orsolya Flasko, Tamas Juhasz, Abel Molnar, Norbert Nemeth

**Affiliations:** 1 Department of Surgery, Faculty of Medicine, University of Debrecen, Moricz Zsigmond Str. 22, H-4032 Debrecen, Hungary; kincses.gergo@med.unideb.hu; 2 Department of Operative Techniques and Surgical Research, Faculty of Medicine, University of Debrecen, Moricz Zsigmond Str. 22, H-4032 Debrecen, Hungary; fazekas.laszlo@med.unideb.hu (L.A.F.); varga.adam@med.unideb.hu (A.V.); matrai.adam@med.unideb.hu (A.A.M.); xuan.loc.nguyen@unidebhu.onmicrosoft.com (N.X.L.); barabasikincso@mailbox.unideb.hu (K.B.); 3 Department of Otorhinolaryngology and Head and Neck Surgery, Faculty of Medicine, University of Debrecen, Nagyerdei Str. 98., H-4032 Debrecen, Hungary; flasko.anna.orsolya@med.unideb.hu; 4 Department of Anatomy, Histology and Embriology, Faculty of Medicine, University of Debrecen, Nagyerdei Str. 98., H-4032 Debrecen, Hungary; juhaszt@anat.med.unideb.hu; 5 Department of Dermatology, Faculty of Medicine, University of Debrecen, Nagyerdei Str. 98., H-4032 Debrecen, Hungary; molnar.abel@med.unideb.hu

**Keywords:** musculocutaneous flap, microcirculation, incident dark field imaging technique, hemorheology, tensile strength, histology

## Abstract

In reconstructive surgery, usage of different flaps is essential to cover tissue defects. Twisting, stretching or damaging the vascular pedicle may jeopardize the flaps’ viability. The aim of our experiment was to monitor tissue perfusion parameters of local versus rotated musculocutaneous flaps. In rats, musculus cutaneus maximus-based muscle–skin flaps were prepared bilaterally: one was sutured back to its original position, while the other flap was rotated to the ventral chest region (Flap group). In the Control group, flaps were not prepared. Tissue microcirculation was monitored intraoperatively, and on the 7th and 14th postoperative days. Blood samples were taken for testing hematological and hemorheological parameters. At the end of the observation period, biopsies were taken for biomechanical (tensile strengths) and histological investigations. We found that leukocyte and platelet counts significantly increased in the Flap group, while erythrocyte deformability decreased and aggregation increased. Although both local and rotated flaps survived and wound healing progressed well, in microcirculatory recordings, hypoperfusion and visible red blood cell aggregates were seen mostly in the rotated flaps. The rotated flaps were biomechanically weaker compared to local flaps or intact skin regions. This new model seems to be suitable for studying further flap pathophysiology focusing on tissue perfusion.

## 1. Introduction

Invasive breast cancer is the second most common and, in the case of women, the leading neoplasia worldwide according to the WHO data. In early-stage cases, breast conserving surgery is possible, but mastectomy still has a significant role [1,2,3,4]. In most cases, closing the wound is not a problem after mastectomy. If an extensive, advanced stage tumor is operated on, the closure of the skin could be challenging due to the extensive skin and soft tissue defect [2,4,5]. In this situation, applying different types of flaps could be necessary. Poor condition of the remaining tissues (irradiation, inflammation, revascularized region) also could be an indication for tissue transfer [3,4,6]. Besides surgical indications, reconstruction has significant quality-of-life benefits in many patients undergoing mastectomy. There are several ways of reconstruction with either local, transpositioned (e.g., rotated) or transferred free flaps using microvascular anastomoses [4,7,8]. Pedicled flaps, unlike grafts, remain attached to a vascular supply known as a pedicle. Rotated flaps pivot adjacent tissue around an axis to close a primary defect, essentially rotating skin into the defect [7,8].

Necrosis of the flap can be decreased effectively by applying a personalized modified technique for specific patients [2,3,9,10,11]. Nowadays, parallel to the dynamic development of microsurgical techniques and devices, free flap transplantation is gaining attention. In the past few decades, it has been commonly applied in breast, head, and neck and lower limb reconstruction [2,6,12,13]. Preserving the proper blood supply is an essential criterion for successful transposition and transplantation. Impaired perfusion lengthens the healing period, increases the chance of suppuration, abscess formation, and necrosis, and in severe cases can lead to flap failure [11,14,15].

For detecting the possible flap damage, monitoring methods have been developed [12,15,16,17,18,19,20]. Clinical signs, examinations, such as evaluation of color, tissue temperature change and capillary refill, and various flap perfusion monitoring possibilities are critical components of postoperative follow-up; nevertheless, they greatly rely on the experience and the judgment of the observer [18,19,21,22]. Laboratory parameters like red blood cell deformability and red blood cell aggregation have great importance. These parameters show changes in numerous pathophysiological conditions; therefore, the examination of these parameters is important in surgical and microsurgical experiments as well. Thrombotic and ischemia–reperfusion complications can happen in every phase of reconstructive surgical interventions: during the preparation, the transposition or rotation of the flap, and even during the healing period, which may result in flap failure and necrosis [12,21,22,23]. However, their micro-rheological relations have not been completely revealed yet. Furthermore, we did not find examples in the literature concerning simultaneously investigating micro-rheology and microcirculation, together with histological or biomechanical data comparing local versus rotated flaps.

Our hypothesis was that micro-rheological and microcirculatory parameters together with histological and biomechanical properties can be informative in comparison of various flap positioning and in following up the wound healing process in the early postoperative period. The aim of this study was to investigate local and rotated (transpositioned) musculocutaneous flaps and their remodeling using these methods during the inflammatory and early granulation phase of wound healing.

## 2. Materials and Methods

### 2.1. Experimental Animals and Operative Protocol

The study was approved and registered with the University of Debrecen Committee of Animal Welfare and the National Food Chain Safety Office (registration number: 19/2022/UDCAW), in compliance with national regulations (Act XXVIII of 1998 on the Protection and Humane Treatment of Animals) and EU directives (2010/63/EU).

Sixteen adult male Wistar rats (Crl: WI, bodyweight: 382.99 ± 37.23 g, Toxi-Coop Zrt., Budapest, Hungary) were utilized in the study. The animals were kept in the Department’s conventional-status animal facility (standard cages: Eurostandard IV, Tecniplast, Buguggiate, Italy; temperature: 22 ± 2 °C; humidity: 55 ± 10%; lighting: 12–12 h light/dark cycle). They had free access to standard rat food (SAFE^®^ D132, Complete Care Competence, Augy, France) and water. The animals were involved in the experiments after a 2-week acclimatization period.

For general anesthesia, ketamine hydrochloride (100 mg/bwkg, i.p., CP ketamine hydrochloride 10%, Produlab Pharma BV, Raamsdonksveer, The Netherlands) and xylazine hydrochloride (10 mg/bwkg, i.p., CP xylazine hydrochloride, 2%; Produlab Pharma BV, Raamsdonksveer, The Netherlands) was used [24,25]. Animals breathed spontaneously. To control anesthesia, we observed respiratory rate and depth, color of the mucous membrane and extremities, and a heating pad was used to maintain body temperature during the operation.

Before starting flap preparation, after depilation, we estimated the extension of the angiosome of the lateral thoracic artery and formed a circular-sheet plastic template (area: 7.13 cm^2^) to standardize the operation. Animals were randomly subjected to the Control (sham-operated) group (n = 8) and the Flap group (n = 8) (randomization method: computer-generated random numbers using individual animal identification codes).

**Figure 1:**
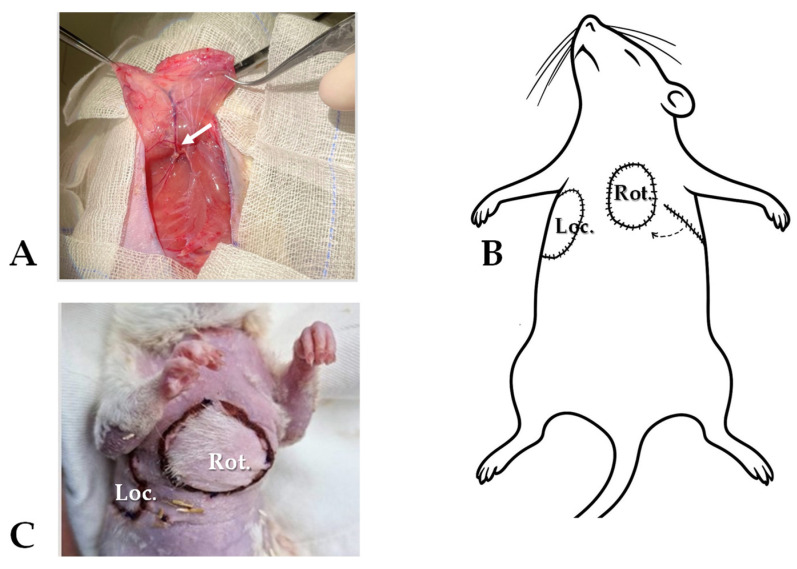
Surgical protocol: (**A**) the musculocutaneous flap preparation (m. cutaneous maximus, pedicled on lateral thoracic vessels—arrow), (**B**) right-side flap resutured to its original position (Loc.), left-side flap rotated (Rot., through a subcutaneously formed tunnel), (**C**) and the pictures of the locally repositioned and rotated, sutured flaps.

In the Control group, depilation above the flap territories—being relevant to the Flap group—anesthesia, immobilization for the time of the operation happened. In the Flap group we marked the skin with the template around the flap territories. After skin disinfection and incision, m. cutaneous maximus-based muscle–skin flaps were prepared bilaterally. The convexity of the flaps looked toward the back and the cranial apex positioned in the frontal part of the armpit. The right-side flap—after elevation—was sutured back to its original position with tension-free anchoraging, simple interrupted stitches and intracutaneous sutures in between (4-0, cutting needle, Silk; SMI, Steinerberg, Belgium). The left-side was rotated to the ventral chest region, where an iatrogenic soft tissue defect was made. Under the connecting skin bridge, we formed a tunnel for the flap rotation. Pulling through the tunnel, the flap was then in its final position and was sutured. The donor site was also sutured (Figure 1). The tunnel preparation was as wide as possible to prevent the strangulation of the supplying vessels.

For postoperative analgesia, Tramadol (15 mg/bwkg/day) was administered for the first three days [26]. Postoperatively, the animals were kept individually and received a plastic collar to prevent autophagy. Daily wound inspection was provided.

Before and after surgery, and on the 7th and 14th postoperative days, blood samples were collected from the lateral tail vein (0.4 mL/each; 26 G Neoflon™ Pro IV Cannula; K3-EDTA, Vacutainer^®^; both: Becton Dickinson GmbH, Franklin Lakes, NJ, USA) to test hematological and micro-rheological parameters.

**Figure 2:**
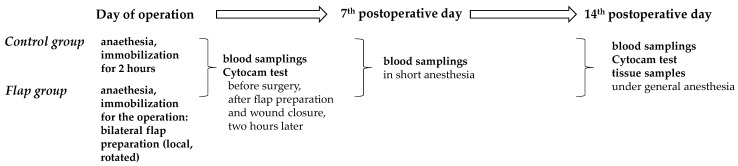
Flow chart of the experimental timeline of the Control and Flap groups with main assessment points.

A summary of the experimental timeline and assessment points are simplified in Figure 2.

### 2.2. Laboratory Techniques

Hematological variables were determined by a Sysmex K-4500 microcell counter (TOA Medical Electronics Co., Ltd., Kobe, Japan). We analyzed white blood cell count (WBC [10^9^/L]), red blood cell count (RBC [10^12^/L]), hematocrit (Hct [%]), platelet count (Plt [10^9^/L]), hemoglobin concentration (Hbg [g/dL]), mean corpuscular volume (MCV [fL]), mean corpuscular hemoglobin content (MCH [pg]), and mean corpuscular hemoglobin concentration (MCHC [g/dL]).

A LoRRca MaxSis Osmoscan ektacytometer (RR Mechatronics International B.V., Zwaag, The Netherlands) was used to test red blood cell deformability, determining the elongation index (EI) in the function of shear stress (SS [Pa]), range: 0.3–30 Pa [27,28]. For the test, 10 μL whole blood was mixed with 2 mL of polyvinylpyrrolidone (PVP)–PBS solution (PVP: 360 kDa, Sigma-Aldrich Co., St. Louis, MO, USA; PVP-PBS solution viscosity = 30–35 mPas, osmolality = 290–310 mOsmol/kg, pH = 7.5). Comparative data from the EI–SS curves were calculated, including EI values at 3 Pa, maximal elongation index (EI_max_), and shear stress at half EI_max_ (SS_1/2_, Pa) by Lineweaver–Burk equation (1/EI = SS_1/2_/EI_max_ × 1/SS + 1/EI_max_) [Baskurt]. Red blood cell deformability impairment is reflected by lower EI or EI_max_, and higher SS_1/2_ values [28,29].

A Myrenne MA-1 erythrocyte aggregometer (Myrenne GmbH, Roetgen, Germany) was used to test red blood cell aggregation based on the light-transmittance method. The test used 20 µL of blood. Light transmission was assessed for 5 or 10 s under static conditions (M 5s and M 10s values, shear rate: 0 s^−1^) or at a low shear rate (M1 5s and M1 10s values, shear rate: 3 s^−1^) [27]. Higher index values indicate enhanced red blood cell aggregation [27,28].

### 2.3. Microcriculatory Measurements

Microcirculation was monitored using a CytoCam-IDF camera (Braedius Medical, Huizen, The Netherlands) [30,31] before surgery, after flap preparation and wound closure, two hours later, and finally, two weeks postoperatively. This imaging technique enables real-time observation of microcirculation. It is based on the Incident Dark Field (IDF) illumination technology, where the device emits green laser light pulses at a wavelength of 540 nm. The short light pulses allow for more accurate imaging of fast-moving red blood cells, while optical resolution exceeds 300 lines/mm, producing sharp contrast images allowing distinction between capillaries and venules. During the offline analysis of the recordings (CytoCamTools V3 Bedside Manager, Braedius Medical, Huizen, The Netherlands), the key parameters included the microvascular flow index (MFI: qualitative scoring of flow velocity [au]), the proportion of perfused vessels (PPV: percentage of vessels with continuous flow [%]), perfused vessel density (PVD: density of vessels actively perfused with flowing blood [mm/mm^2^]), and flow velocity within the vessels. The device can also determine total capillary length and density, total vessel density (TVD: microvascular length/mm^2^ of tissue surface), the number of capillary segments, average capillary length, perfused capillary density, and their proportion [30,31,32].

To standardize video recording with the Cytocam-IDF camera, consistent measurement sites were selected and precise focus adjustments were applied during recording (±2 µm). Short (~3 s) clips were obtained with proper illumination, stable positioning, and minimal pressure; the camera was mounted in a custom-made holder during measurements. Approximately three videos were captured per site, with low-quality clips discarded after scoring for illumination, focus, stability, and artifacts. Recordings were analyzed offline with dedicated software, using consistent algorithms in a blinded and anonymized manner. Resolution and field of view were kept constant (720 × 580 px, 0.94 × 0.75 mm). The pen-like design aided stability, and flow indices such as MFI were assessed by scoring each quadrant [31,32].

### 2.4. Tensile Strength Measurements

During the tensile strength tests, performed on a custom-developed device [33], we used skin strips of uniform width (5 mm) on the 14th postoperative day. These samples included both the intact and operated sections, as well as the healed suture line in the middle. The samples were placed in a tensile testing machine, where a gradually increasing pulling force was applied until they ruptured. The maximum force the samples could withstand before breaking indicates the tensile strength of the tissue. The force required for rupture and elongation were continuously recorded, and the stress–strain curve generated by the machine allowed us to determine the maximum stress, the breaking point, and the slope of the curves [33].

### 2.5. Histological Analysis

On the 14th postoperative day, animals were anesthetized, and we collected standard-sized and shaped samples from both the control and rotated flaps for histological examination. These samples included intact tissue, flap tissue, and the transitional zone where the tissues anastomized. The samples were placed in formalin and then processed using conventional methods for embedding and sectioning. Serial sections were prepared, and staining was performed using haematoxylin-eosin (H&E, Sigma-Aldrich, St. Louis, MO, USA) for morphological analysis and orcein (Sigma-Aldrich, St. Louis, MO, USA) for elastin visualization.

All staining procedures followed the manufacturer’s protocols. Photomicrographs were captured using a DP74 camera (Olympus Corporation, Tokyo, Japan) mounted on an Olympus B×53 microscope (Olympus Corporation, Tokyo, Japan). To examine the orientation of collagen fibers in the vessels, Picrosirius red staining (Sigma-Aldrich, St. Louis, MO, USA) was applied. The samples were analyzed under polarized light using an Olympus B×53 polarization microscope (Olympus Corporation, Tokyo, Japan), with the light plane rotated using λ/4 samples. For measuring the thickness of the epidermis, photomicrographs of H&E-stained sections at 20× magnification were analyzed using ImageJ 1.40 g freeware. Twenty independent measurements were taken from each slide, and six independent skin strips were examined per experimental group. In orcein-stained sections, the normal color was inverted to green and black to enhance pixel contrast. The green pixel count was quantified using ImageJ freeware.

### 2.6. Statistical Analysis

The Mead’s resource equation method was employed to determine the required sample size for the experiment [34]. Statistical analyses were conducted using SigmaStat Software 3.1.1.0 (Systat Software Inc., San Jose, CA, USA). Descriptive statistics are presented as means ± standard deviation (S.D.). Data with normal distribution underwent a *t*-test, while data without normal distribution were analyzed using the Mann–Whitney rank sum test for inter-group comparison. One-way ANOVA or Kruskal–Wallis test was used for intra-group comparison. For multiple comparison, Bonferroni’s test was also applied to all tests where multiple statistical tests or comparisons were performed simultaneously. A value of *p* < 0.05 was considered statistically significant.

## 3. Results

### 3.1. General Observations

We did not see thrombotic complications, swelling or extended flap necrosis. The healing process of the sutured wounds turned well into the granulation phase. We observed partial, thin marginal necrosis only in 2 cases out of the 16 flaps (1 in a local and 1 in a rotated flap). These cases were not included in the final data analyses.

### 3.2. Changes of Hematological Parameters

Hematological parameters reflected the acute phase reactions during the inflammatory and early granulation phase of the wound healing during the 2-week observation period. White blood cell and platelet counts increased by the 1st and 2nd postoperative weeks, in a larger manner in the Flap group (WBC on 7th day: *p* = 0.013 and 14th day: *p* < 0.001; Plt on 7th day: *p* < 0.001 and 14th day: *p* = 0.002 vs. base). RBC count, hemoglobin concentration and hematocrit decreased more in the Flap group (all: *p* < 0.001) (Table 1).

### 3.3. Alterations in Red Blood Cell Deformability

Red blood cells’ elongation index at 3 Pa shear stress decreased by the 7th postoperative day, together with the calculated EI_max_ values (*p* < 0.001) and a slight increase in SS_1/2_, all reflecting moderately impaired erythrocyte deformability in Flap group. Their ratio values (EI_max_/SS_1/2_) showed an increase by the end of the observation period (Table 2).

### 3.4. Red Blood Cell Aggregation Changes

**Figure 3:**
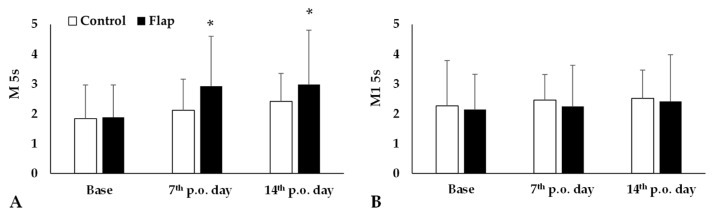
Red blood cell aggregation index values M 5s (**A**) and M1 5s (**B**) in the Control and Flap groups before operation (base), on the 7th and 14th postoperative days. Means ± S.D., * *p* < 0.05 vs. base. For multiple comparison Bonferroni’s test was also applied.

Red blood cell aggregation index value M 5s significantly increased by the 7th and 14th postoperative days (both: *p* < 0.001, post hoc power analysis: 7th p.o. day: 32.1% and 14th p.o. day: 31.2%) in the Flap group, while the values of Control group did not show important alterations. Other index values, such as M1 5s did not change significantly (Figure 3).

### 3.5. Microcirculation

**Figure 4:**
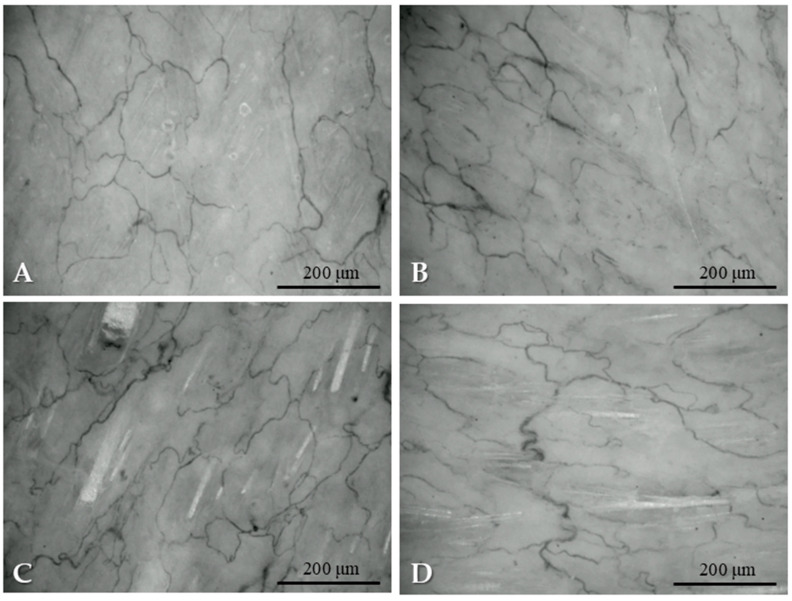
Representative images of microcirculation captured from the IDF-videomicroscopy recordings: before flap preparation (**A**), after preparation (**B**), after suturing the flap (**C**), on the 14th postoperative day (**D**).

Analyzing the videomicroscopy recordings, we observed that in the initial phase of the operation the distribution of the blood vessels was regular. There was no microcirculary or vessel aberration. Following surgical preparation, the microcirculation moderately and temporarily impaired, and hypoperfused areas appeared. After suturing, the microcirculation normalized. On the 14th postoperative day there were some hypoperfused areas, enlarged vessels and red blood cell aggregates dominantly in the rotated flaps (Figure 4).

**Figure 5:**
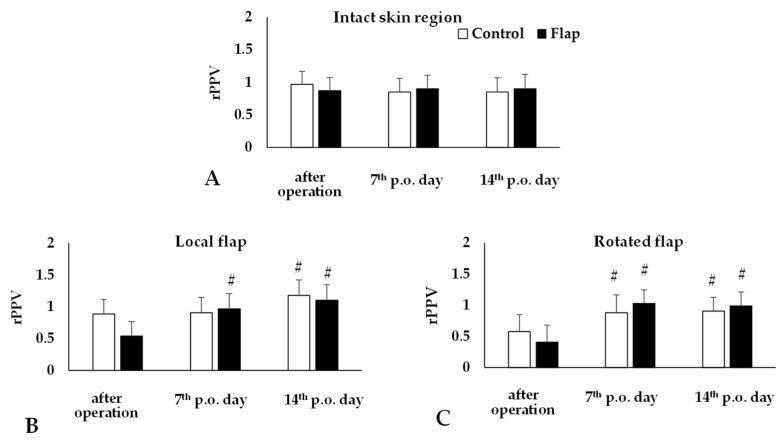
Changes of proportion of perfused vessels relative to their base (rPPV) on intact abdominal skin region (**A**) and in the local (**B**) and rotated (**C**) flaps of Control and Flap groups. Means ± S.D., # *p* < 0.05 vs. after operation. For multiple comparison Bonferroni’s test was also applied.

The recordings were analyzed by the software, providing numerical data. The most obvious alterations were seen in the changes of proportion of perfused vessels compared to base values. In the intact abdominal skin region there was no change, while both in the local and rotated flaps, a significant decrease was seen just after operation (rotated flap: *p* = 0.002 vs. intact skin region), and values increased by the 7th (rotated flap: *p* = 0.014) and 14th postoperative days (rotated flap: *p* = 0.004) (Figure 5). The results of the post hoc power analysis were the following in the case of local flap: Control p.o. 14th day: 69.5%; Flap p.o. 7th day: 95.2%, p.o. 14th day: 99.7%, while in the case of rotated flap: Control p.o. 7th day: 59%, p.o. 14th day: 75.3%, Flap p.o. 7th day: 99.9%, p.o. 14th day: 99.6%.

### 3.6. Tensile Strenght

During tensile strength measurements, the rotated groups showed significantly weakened tissues. The elasticity and functionality also decreased in the rotated group. On the 14th postoperative day, the intact skin maximal tensile strength was much higher than in the local or rotated flaps (both: *p* < 0.001). In case of flap rotation, the tissue strength decreased in a larger magnitude. In parallel, the slope of the force–stretching curve was significantly lower compared to intact skin region (local flap: *p* = 0.021; rotated flap: *p* = 0.002) (Table 3).

### 3.7. Histological Alterations

**Figure 6:**
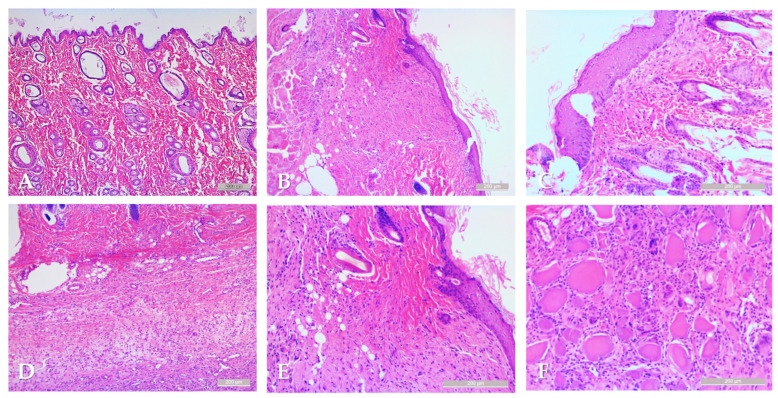
Representative histological sections: intact skin region (**A**), rotated flap with granulation tissue in the suture region (**B**), local flap (**C**), granulation tissue along the flap basement (**D**), granulation and intact tissue border of rotated flap (**E**), foreign body type giant cells in a local flap (**F**). Hematoxylin–Eosin staining, scale bar: 200 μm.

Samples from the intact skin region did not show any histological alterations. In the case of rotated flaps, granulation tissues appeared in the incision area, as a normal part of the healing process. If the wound edges did not fit properly, excessive scar tissue occurred with increased neovascularization, and larger number of fibroblasts and inflammation cells. On the basement of the flap, the granulation tissues also appeared, filling the tissue gaps. Granulation tissues appeared not only in the incision region, but also around the flaps. The border between the intact and granulation tissues was clearly recognizable. In some slices, due to the sutures, some foreign-body-type giant cells (fusion of the macrophages) also appeared (Figure 6).

## 4. Discussion

There are several (flap damaging) risk factors that influence the healing process and might cause damage to the flaps. Numerous studies investigate these factors. Bekara et al. stated that elderly age, diabetes, and arteriopathy are significant risks for flap complications in the lower extremity [13]. Gong et al. found that soft tissue defect site, flap size and postoperative wound infection are increasing the risk of pedicled flap necrosis in hand soft tissue defect reconstruction [10]. Mlodinow et al. revealed that smoking status and increased age are also important risk factors [11]. Qiu et al. reported a high number of risk factors like injury reason, length–width ratio of wound, thickness of pedicle, operation time, injury site, direction of blood perfusion in the flap and operating methods, rather than age, sex and size of flap. Minimizing or eliminating these factors provides a lower chance of flap failure [35].

For studying flap pathophysiology, numerous animal models are known [36,37,38,39,40,41]. In this study, continuing our previous research topic [38,40,41], we used and refined a musculocutaneous flap model, based on cutaneous maximus muscle [38] and pedicled on the lateral thoracic artery and vein [42,43]. The novelty of this study is the usage of rotated flaps, which have more clinical relevance than investigating local flaps (e.g., prepared, elevated and re-sutured) only. In this setting we could investigate the hypothesized different perfusion patterns of the two flap types in the same body, being associated with systemic micro-rheological alterations of circulating blood.

The cutaneus maximus-based muscle–skin peduncle flap supplied by a relatively long vessel branch gives an opportunity for farer transpositions. In our experiment we simulated a distant option. The formation of a subcutaneous tunnel is essential to avoid unnecessary invasivity. The tunnel size should always be wide enough to prevent strangulation of the supplying vessel. During the operations we paid attention to creating an appropriate tunnel to prevent compression or distortion of the flap pedicle containing the supply in the vessels. During the preparation we observed that complete dissection of the cutaneal muscle can cause slight venous congestion on the donor site. Muscle resection around the bean-shaped flap can decrease this effect and provide better flap survival as well. Investigating the effect of rotation on muscle functions could be an interesting question for further studies.

The result of this study showed that there are significant hematological and micro-rheological alterations in the early healing phase of local and rotated musculocutaneous flaps in rat models. The observed deterioration of red blood cell deformability and aggregation, along with changes in hematocrit and white blood cell count, supports the impact of the ischemia–reperfusion effect on microcirculation of the flaps [38,41]. These findings align with previous research about the microcirculatory alterations in flap ischemia, which also suggest that red blood cell deformability and aggregation are significantly impaired in the early postoperative period, leading to potential thrombotic complications and flap failure [38,40,41,44]. In this model we could demonstrate microcirculatory differences in comparison of local and rotated flaps, while simultaneously investigating the micro-rheological features of circulating blood. The enhanced red blood cell aggregation was associated with different microcirculatory patterns in the two flap types, suggesting that the new pedicle vessel position in the rotated flaps and the supposedly altered flow dynamics together might have an effect of flap perfusion. Since the real, in vivo impact of altered micro-rheology on microcirculation has not been fully elucidated yet, these observations may provide further data on the factors influencing tissue perfusion.

It has been previously shown that acute phase reaction and inflammatory processes, as well as metabolic alterations and free-radical damage, various drugs and agents (e.g., anticoagulants, contrast materials, vasodilatative agents, anti-inflammatory and anti-platelet drugs, etc.) may modulate micro-rheological parameters, often resulting in impaired red blood cell deformability and enhanced erythrocyte aggregation [44,45]. These may lead to further microcirculatory deterioration. However, these kinds of special micro-rheological investigations are still not routinely used in clinical laboratories, as these devices are dominantly used for research work (clinical and experimental) [27,28].

During reconstructive surgery, choosing the proper type and size of the flap is essential in the aspect of the vasculature as well [1,6,46]. To prevent the pedicle torquation and damage, atraumatic preparation and tissue handling is indispensable [6,7,13]. In our study we used a standard-sized and shaped musculocutaneous flap type based on cutaneus maximus muscle and lateral thoracal vessels in its pedicle. The proper vessel length is pivotal for the flap to maintain sufficient blood supply without stretching, elongating, torquating or significantly altering the vessel geometry [1,13,17,47,48,49]. These factors might influence the wound healing process and tissue regeneration. Lu et al. compared the tensile strength of the tissues with bFGF and the intact skin. They found that the treated skin regained its elasticity and strength, but the difference was not significant [50]. Although we did not use any agents for enhancing the tissue regeneration, there was significant difference between the two flap types. The transpositioned flaps were biomechanically weaker with lower elasticity. It is supposed that stretching of the flap pedicle in the subcutaneous tissue tunnel may influence the perfusion while the animal is moving, and thus, the wound healing process can be influenced as well.

The main limitations of the study include the limited case number (n = 8 per group) and the relatively short follow-up period that did not allow conclusions about long-term biomechanical and tissue perfusion outcomes. The exclusion of two necrotic flap cases reduced further the evaluated case number; however, according to the sample size calculation, it was still tolerable for this kind of animal experiment in accordance with the 3Rs Principles. The experiment included comparison of flaps within the same animal, and we have to count that even vascular variability may occur. The investigative methods we used also have technical limitations (e.g., resolution, tissue area and depth, variability of parameters, power of tests, accuracy/relevance of off-line calculated numerical parameters, etc.), as many other microcirculation monitoring devices (e.g., laser Doppler flowmetry, idocyanid-green or fluorescence angiography, laser speckle contrast analysis, near infrared spectroscopy, etc.) are known with more or less technical limitations [51,52]. However, the used IDF technique provided stable microcirculatory recordings with numerous off-line analyzed parameters, reducing observer-dependent variability. Furthermore, inter-species differences in microvasculature, blood macro- and micro-rheology [44], physiology of animals and the anatomical differences can also be listed. In humans, there is no m. cutaneous maximus; however, the model resembles a stronger fasciocutaneous flap [36]. Therefore, this interspecies anatomical difference is another factor limiting direct translation of the results.

## 5. Conclusions

In this descriptive study, we demonstrated that micro-rheological parameters (red blood cell deformability and aggregation) together with hematological parameters have an effect during the early wound healing phase of local and rotated musculocutaneous flaps. The alterations reflected acute phase reactions. Comparing the two flap types, microcirculatory parameters did not worsen in rotated flaps; however, the values were lower compared to local flaps.

Although both local and rotated flaps survived and wound healing progressed well, in microcirculatory recordings, hypoperfusion and visible red blood cell aggregates were seen mostly in the rotated flaps. The rotated flaps were biomechanically weaker compared to local flaps or intact skin regions.

The applied model seems to be suitable for studying further flap pathophysiology focusing on tissue perfusion and preventing complications (e.g., optimizing the flap size versus vascular pedicle, various tension-free techniques, flap preconditioning, methods/agents for facilitating wound healing).

## Figures and Tables

**Table 1:** Alterations of hematological parameters in the Control and Flap groups before operation (base), and on the 7th and 14th postoperative (p.o.) days.

Variable	Group	Base	7th p.o. Day	14th p.o. Day
WBC [10^9^/L]	Control	11.05 ± 2.2	11.9 ± 3.0	13.64 ± 4.7
	Flap	9.95 ± 4.1	12.78 ± 2.9 * (35.7%)	15.65 ± 3.5 * (84.9%)
RBC [10^12^/L]	Control	8.32 ± 0.6	7.56 ± 0.7 * (64.5%)	8.23 ± 0.4
	Flap	7.75 ± 0.5	7.19 ± 0.7 * (45.3%)	6.7 ± 0.7 * (93.2%) # (100%)
Hgb [g/dL]	Control	15.76 ± 0.8	14.36 ± 1.3 * (73.7%)	15.22 ± 0.8 * (27.1%)
	Flap	14.88 ± 0.9	13.69 ± 1.4 * (52.5%)	12.63 ± 1.2 * (98.9%) # (99.9%)
Htc [%]	Control	46.92 ± 3.1	42.93 ± 4.6 * (53%)	45.99 ± 2.0
	Flap	44.35 ± 2.3	40.31 ± 3.8 * (73%)	38.39 ± 3.7 * (97.2%) # (99.9%)
MCV [fL]	Control	56.38 ± 1.3	56.7 ± 1.9	55.92 ± 1.4
	Flap	57.12 ± 2.6	56.07 ± 0.8	57.02 ± 3.5
MCH [pg]	Control	18.95 ± 0.7	19.01 ± 0.4	18.51 ± 0.6
	Flap	19.18 ± 0.62	19.04 ± 0.7	18.92 ± 1.2
MCHC [g/L]	Control	33.63 ± 0.8	33.53 ± 1.0	33.09 ± 0.5
	Flap	33.56 ± 1.0	33.97 ± 1.4	32.94 ± 1.0
Plt [10^9^/L]	Control	820.38 ± 133.9	997.43 ± 177.14 * (61.6%)	931.9 ± 232.6
	Flap	764 ± 94.0	978.53 ± 150.6 * (92.8%)	911.8 ± 198.1 * (47.9%)

mean ± SD; * *p* < 0.05 vs. base, # *p* < 0.05 vs. Control; WBC: white blood cell count; RBC: red blood cell count; Hgb: hemoglobin concentration; Hct: hematocrit; MCV: mean corpuscular volume; MCH: mean corpuscular hemoglobin; MCHC: mean corpuscular Hgb; Plt: platelet count; for multiple comparison Bonferroni’s test was also applied. Post hoc power analysis results are in brackets.

**Table 2:** Alterations of red blood cell deformability describing parameters in the Control and Flap groups before operation (base), and on the 7th and 14th postoperative (p.o.) days.

Variable	Group	Base	7th p.o. Day	14th p.o. Day
EI at 3 Pa [au]	Control	0.327 ± 0.03	0.334 ± 0.02	0.347 ± 0.03 * (26.5%)
	Flap	0.327 ± 0.03	0.313 ± 0.04	0.336 ± 0.02
EI_max_ [au]	Control	0.540 ± 0.03	0.539 ± 0.02	0.558 ± 0.01 * (36.3%)
	Flap	0.552 ± 0.02	0.525 ± 0.03 * (56.3%)	0.545 ± 0.03
SS_1/2_ [Pa]	Control	2.16 ± 0.6	1.89 ± 0.3	1.87 ± 0.4
	Flap	2.18 ± 0.6	2.22 ± 0.9	1.90 ± 0.3
EI_max_/SS_1/2_ [Pa^−1^]	Control	0.27 ± 0.07	0.228 ± 0.04	0.311 ± 0.06 * (24.1%)
	Flap	0.26 ± 0.06	0.259 ± 0.07	0.293 ± 0.04

mean ± SD; * *p* < 0.05 vs. base; EI at 3 Pa: elongation index at shear stress of 3 Pa; EI_max_: maximal elongation index; SS_1/2_: shear stress at half EI_max_; for multiple comparison Bonferroni’s test was also applied. Post hoc power analysis results are in brackets.

**Table 3:** Tensile strength and force–stretching curve slope values of intact abdominal skin region, local and rotated flaps tested on tissue samples taken on the 14th postoperative day.

Region/Flap	Tensile Strengh [N]	Slope of Curve
Intact abdominal skin	19.97 ± 5.61	0.157 ± 0.115
Local flap	2.98 ± 0.87 * (100%)	0.044 ± 0.03 * (76.7%)
Rotated flap	2.44 ± 0.58 * (100%)	0.024 ± 0.006 * (90.4%)

mean ± SD; * *p* < 0.05 vs. intact abdominal skin region; Post hoc power analysis results are in brackets.

## Data Availability

The data presented in this study are available on request from the corresponding author. The data are not publicly available due to ethical constraints.
